# The 6th Russian School of Colorectal Surgery meeting: 5–6 April 2012, Moscow, Russia

**DOI:** 10.1007/s10151-012-0841-6

**Published:** 2012-05-17

**Authors:** N. Stojanovic, M. Janic

**Affiliations:** General Hospital, Pancevo, Serbia

The capital of Russia these days is a metropolis with 12 million people. Two million people daily enter and leave Moscow!

The last time we participated in this meeting was 3 years ago. Our first impression this time was that the meeting had tripled in size under the leadership of Prof. V.P. Tsarkov. He and his team spread their arms and gave a warm welcome to more than 1,500 participants.

In the early spring of this year,for the sixth time, the Russian School of Colorectal Surgery offered a very interesting and busy program. The conference was divided into 2 days and two important and fascinating topics. The first day was dedicated to benign pathology of the rectum and pelvic floor. Hemorrhoidal disease was a popular topic for live surgery and problem solving. G. Millito, M. Janic, and S. Regadas demonstrated hemorrhoidectomy with Ligasure, hemorrhoidectomy with monopolar electrocautery, and stapled hemorrhoidopexy. It was concluded that surgical hemorrhoidectomy should be reserved for patients who do not respond to outpatient treatment and those who have large external hemorrhoids, or combined internal and external hemorrhoids with significant prolapse (grades III–IV).

E. Rullier performed laparoscopic ventral rectosacropexy for descending perineum syndrome with new mesh and a new method for securing it. This session was chaired by M. Pescatori and moderated by V.P. Tsarkov, I. Nechai, D. Khubezov, and V. Egiev, all of whom stimulated the discussion with their comments.

The following session consisted of lectures on another interesting topic: diagnosis and treatment of pelvic floor dysfunctions. With his fascinating presentation, M. Pescatori opened another perspective on this problem and spoke about the iceberg syndrome and treatment solutions. S. Regadas gave a masterly lecture based on several years of experience with a novel 3-D dynamic anorectal ultrasoound technique for the assessment of perineal descent. A.A. Popov made an important contribution with his talk on pelvic floor descent from a gynecologist’s point of view. The session closed with an interesting lecture by J. Korcek on transvaginal procedures in the treatment of rectocele and pelvic floor syndrome.

The next day in the Congress Hall of the Hotel Radisson Slavyanskaya, the seats were rapidly taken and even standing room was at a premium as the hall filled with curious surgeons.

Live surgery began in two operating theatres simultaneously. R. Bergamaschi, A. Parvajz, and V.P. Tsarkov masterfully demonstrated their techniques in laparoscopic and open colectomy. The next session was devoted to new perspectives in the treatment of colon cancer. The series of lectures began with R. Bergamaschi examining the issue of laparoscopic colon cancer surgery as a new standard of care, followed by E. Rullier speaking about issues in laparoscopic treatment of colon cancer and then E. Tiret, who covered the current standards of care. C.G. Fu presented an impressive case series of hand-assisted laparoscopic surgery. The session closed with a very interesting lecture by K. Maeda on the pros and cons of lymph node dissection in colon cancer.

The last session was dedicated to various themes: from a surgical view of colon cancer metastases, presented by V.P. Tzarkov, through questions about when and how to treat dehiscence of colonic anastomoses, discussed by Y. Panis. A. Zakharenko described some very complicated cases of colon cancer. A. Parvajz stressed the importance of a training program for surgeons in the colorectal field. A young yet very experienced hepaticobiliary surgeon from Liverpool, S. Fenwick, gave an exciting talk on new hope for the treatment of liver metastatic colorectal cancer.

An impressive master class on the first day was given by S. Regadas, with a helping hand from the staff of the Department of Colorectal and Pelvic Floor Surgery of the Russian National Center of Surgery.

This very successful meeting wound to an end on the evening of the second day, as the Moscow river flowed into the night, quietly singing “Kalinka”.

